# Effect of Water Chemistry on Antimony Removal by Chemical Coagulation: Implications of ζ-Potential and Size of Precipitates

**DOI:** 10.3390/ijms20122945

**Published:** 2019-06-17

**Authors:** Muhammad Ali Inam, Rizwan Khan, Muhammad Akram, Sarfaraz Khan, Ick Tae Yeom

**Affiliations:** 1 Graduate School of Water Resources, Sungkyunkwan University (SKKU) 2066, Suwon 16419, Korea; aliinam@skku.edu (M.A.I.); rizwankhan@skku.edu (R.K.); 2 Shandong Key Laboratory of Water Pollution Control and Resource Reuse, School of Environmental Science and Engineering, Shandong University, Qingdao 266200, China; m.akramsathio@mail.sdu.edu.cn; 3 Key Laboratory of the Three Gorges Reservoir Region Eco-Environment, State Ministry of Education, Chongqing University, Chongqing 400045, China; Sfk.jadoon@yahoo.com

**Keywords:** adsorption, antimony removal, coagulation, ferric chloride, precipitates, water treatment

## Abstract

The process of coagulation and precipitation affect the fate and mobility of antimony (Sb) species in drinking water. Moreover, the solubility and physico-chemical properties of the precipitates may be affected by the media chemistry. Accordingly, the present study aimed to investigate the removal of Sb(III, V) species by ferric chloride coagulation under various water chemistry influences with a particular focus on the role of the properties of the precipitates. The results indicated that the amount of Sb(III) removed increased with increasing solution pH, showing the insignificant effects of the hydrodynamic diameter (HDD) and ζ-potential of the precipitates. However, no Sb(V) removal occurred at alkaline pH values, while a highly negative ζ-potential and the complete dissolution of precipitates were observed in the aqueous solution. The solution pH was also useful in determining the dominant coagulation mechanisms, such as co-precipitation and adsorption. The Fe solubility substantially affects the Sb removal at a certain pH range, while the HDD of the precipitates plays an insignificant role in Sb removal. The presence of divalent cations brings the ζ-potential of the precipitates close to point of zero charge (pzc), thus enhancing the Sb(V) removal at alkaline pH conditions. Pronounced adverse effects of humic acid were observed on Sb removal, ζ-potential and HDD of the precipitates. In general, this study may provide critical information to a wide group of researchers dealing with environmental protection from heavy metal pollution.

## 1. Introduction

Antimony (Sb) and its compounds are ubiquitous in the environment due to natural processes and the consequences of modern industrial activities [1,2]. High levels of Sb pollution, that is, 1000, 239 and 2–6384 µg/L, have been found in water bodies near Sb mines in Slovakia, the Kantishna Hills mining district (Alaska, USA) and Sb mine at Xikuangshan in Hunan Province (China), respectively [3,4,5]. Sb contamination in surface and groundwater has raised global concerns due to its acute toxicity to both humans and the ecosystem [6]. The adverse health effects of the oral uptake of water-soluble Sb in the human body may lead to abdominal cramps, cardiac toxicity, vomiting and diarrhea [7]. Therefore, Sb and its compounds are considered to be a pollutant of priority interest by the United States Environmental Protection Agency (USEPA) and the European Union (EU) [2]. Moreover, the South Korean Ministry of Environment has also included Sb in the monitoring list of the Water Quality and Aquatic Ecosystem Conservation Act [8]. In order to minimize the toxicological risks of Sb to humans, the USEPA, EU, Korea and the World Health Organization (WHO) have set Sb regulatory standards limited to 6, 10, 20 and 5 µg/L respectively [9,10].

The main reported Sb removal techniques include chemical coagulation/adsorption [2,10,11,12,13,14]. It has been shown to be a cost-effective treatment technology for removing heavy metals such as Sb from water [15]. Extensively used commercial coagulants such as iron salts have proven to be more effective in remediating Sb than aluminum salts [10,16]. For instance, the removal of trivalent Sb(III) species never achieved more than 25% using aluminum sulfate across a wide pH range (4–10). Under acidic pH (4.5–6.0), the Sb(V) species showed only 25% removal while negligible removal was observed for other studied pH by aluminum sulfate [10]. In contrast, the ferric chloride (FC) coagulant presented much better removal performance of both Sb species, where more than 80% Sb(III) removal was achieved across a broad pH range. Moreover, more than 90% Sb(V) removal was achieved by FC at acidic pH (4.5–5.5) and then decrease in removal trend was observed with pH [10]. The mechanisms that have been suggested to be responsible for the removal of soluble Sb species include precipitation, co-precipitation and adsorption [10]. However, the possibility of Sb precipitation, that is, the formation of FeSbO_3_ for Sb(III) or FeSbO_4_ for Sb(V) after interaction with Fe(III) species, has been thermodynamically disregarded [10]. By contrast, co-precipitation and adsorption mechanisms are usually used to explain the removal of Sb from the aquatic environment [10,12,13,14]. To date, no study has explored the dominant removal mechanism of Sb during chemical coagulation. Therefore, for efficient water treatment applications, it is crucial to understand the removal behavior of Sb(III, V) species after interaction with ferric salts.

Natural water consists of a variety of constituents that can influence the removal efficiency of the target pollutants. A number of studies [10,12,13,14] have reported that the removal of Sb depends on factors of solution chemistry such as pH, contaminant redox form, ionic strength and organic matter. It has been demonstrated that solution pH has an insignificant effect on Sb(III) removal; however, Sb(V) removal was remarkably decreased at alkaline pH conditions due to the complete dissolution of FC precipitates during the coagulation process [12]. Earlier studies suggested that the sorption of Sb onto precipitated Fe depends on the Sb species type and indicated the higher adsorption capacity of Sb(III) compared to that of Sb(V) species under neutral pH conditions [13]. The presence of metal cations (such as K^+^, Ca^2+^ and Mg^2+^) could also affect the removal of Sb species from water. Previous studies [10,11,14] have also shown the antagonistic effect of organic matter, that is, humic acid (HA) and fulvic acid, on Sb removal during the chemical coagulation process. However, the colloidal properties of FC precipitates are important and should be considered for Sb removal during the water treatment process. To the best of our knowledge, the Sb removal behavior with particular emphasis on colloidal properties and stability of FC precipitates has rarely been explored by researchers in the environmental and chemical fields.

Accordingly, the objective of the current study was to examine the effects of different water chemistry parameters such as pH, metal cations and organic matter on the ζ-potential, hydrodynamic diameter (HDD) and solubility of precipitates and consequently, their effects on Sb(III, V) removal by chemical coagulation. Secondly, the present study aims to investigate the dominant Sb removal mechanism, in order to better elucidate the removal behavior of Sb species during the water treatment process.

## 2. Results and Discussion

### 2.1. Effect of pH on Physico-chemical Properties of Precipitates and Sb Removal

**Figure 1:**
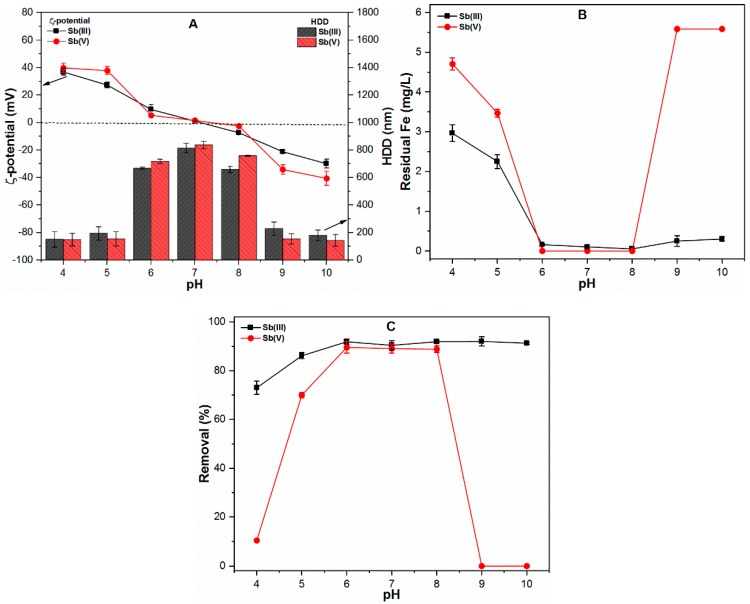
At various pH ranges (4–10), ferric chloride (FC) dose (0.1 mM), temperature (25 ± 1 °C) and Sb(III, V) concentration (1 mg/L) showing (**A**) ζ-potential (mV) and hydrodynamic diameter (HDD) (nm) of precipitates; (**B**) Residual Fe (mg/L); and (**C**) Removal (%) of both Sb species.

Figure 1A shows the ζ-potential and HDD of early formed FC precipitates in the presence of both Sb(III) and Sb(V) species. The HDD of precipitates exists in the colloidal range, i.e., < 1 µm under the influence of Sb(III, V) species across a wide pH range (4–10). The low HDD of precipitates was observed at acidic and alkaline pH conditions, where the ζ-potential was found to be highly positive and negative. For instance, at pH 4 and 10, the HDD and ζ-potential were observed to be around 150.47 and 179.43 nm and around 36.84 and −29.94 mV, respectively, in the case of Sb(III) species, while it was around 148.42 and 143.26 nm and around 39.79 and −40.59 mV, respectively, in the case of Sb(V) species. Notably, at higher pH, i.e., 9 and 10, Sb(V) species impart a significant influence on the properties of precipitates as compared to Sb(III) species. This observation can be explained by the speciation of Fe(III) and Sb(V) species under those pH conditions. The Fe(III) and Sb(V) species exist as Fe(OH)_4_^−^ and Sb(OH)_6_^−^ at alkaline pH (Supplementary: Figure S1A,B), thus imparting more negative charge on the surface of precipitates, therefore might be responsible for decreasing ζ-potential toward a more negative trajectory. However, the Sb(III) species exist as Sb(OH)_3_ (Supplementary: Figure S1C) and thus had an insignificant effect on the ζ-potential of precipitates. The HDD increases significantly in the pH range of 6–8, which may be attributable to the fact that ζ-potential values were near the point of zero charge (pzc) [17]. At pH 7, the largest HDD of precipitates were shown to be 815.61 nm in the case of Sb(III) and 837.19 nm in the case of Sb(V). The early-formed FC precipitates at acidic and alkaline conditions were found to be highly stabilized under the influence of redox Sb species. Following the flocculation process, the FC precipitates could only be aggregated to around 310 nm in diameter under extreme acidic/alkaline conditions (i.e., pH 4 or 10). By contrast, at neutral pH (i.e., 7), the HDD of precipitates were found to be around 3370 nm (in case of Sb(III)) and 3620 nm (in case of Sb(V)). In general, the solution pH and redox Sb species were found to significantly influence the ζ-potential and HDD of FC precipitates.

Figure 1B shows the residual Fe concentration as a function of pH using FC coagulation. The results indicated that residual Fe concentration was not affected significantly in the presence of Sb(III) species at pH greater than 6. By contrast, the Sb(V) species substantially enhanced the Fe solubility, particularly at extremely alkaline conditions (viz. pH 9 and 10). At acidic pH (viz. 4 and 5), a relatively higher residual Fe concentration was observed under the influence of Sb(V) species (Figure 1B). This may be attributed to the fact that the ζ-potential values were more positive at acidic pH and more negative at alkaline pH in the presence of Sb(V) species (Figure 1A). The complete dissolution of FC precipitates under alkaline conditions may also result from the interaction of negatively charged Sb(V) with negatively charged Fe(III) species (Supplementary: Figure S1A,B), where the strong electron transfer from Sb(OH)_6_^−^ to Fe(OH)_4_^−^ provides a pathway for Fe-O bond breakage, thus increasing the residual Fe concentration in the solution [12,13]. It should be noted that almost complete Fe precipitation was observed in the pH range of 6–8 in the presence of both redox Sb species (Figure 1B). Since, Fe species predominantly exist in the form of Fe(OH)_2_⁺, thus interaction of these positively charge Fe species with neutral Sb(OH)_3_ and Sb(OH)_6_ˉ under a pH range of 6–8 might favor the Fe precipitation process (Figure S1). Moreover, such observation could be explained by the fact that precipitates with surface potential below ±15 mV are considered to be unstable under electrostatic interaction and tend to form larger agglomerate in solution [18]. The closeness of pH values to point of zero charge (pzc) of FC precipitates might also be responsible for the destabilization of precipitates, thereby reducing the residual Fe concentration across the pH range (6–8) [17].

The removal efficiencies of both Sb species across the broad pH range of 4–10 are shown in Figure 1C. The results showed the insignificant effect of ζ-potential and HDD of the precipitates on the removal of Sb(III) from water. As shown in Figure 1C, Sb(III) removal increases with increasing pH, since the Fe precipitates increase across a wide pH range (Figure 1B). Moreover, the presence of the first dissociation constant pKa of Sb(OH)_3_ at 10.4 indicates that Sb(III) predominantly exists in the neutral molecular form [2]. Our previous studies also indicated the strong adsorption potential of Sb(III) species onto Fe precipitates regardless of the Fe surface potential, as observed in the current work [12,13]. Compared to Sb(III) removal, the removal of Sb(V) was significantly influenced by the ζ-potential and solubility of FC precipitates (Figure 1C); however, the HDD of precipitates did not directly affect the elimination of Sb from aqueous media. The Sb adsorbed FC precipitates were preferentially removed by a 0.22 µm polyethersulfone (PES) membrane filter. However, in the case where HDD of precipitates was <220 nm (particularly at acidic and alkaline pH), the electrostatic attraction and specific adsorption between colloidal particles and membrane surface might be the removal mechanism of Sb species [17]. The relatively low Sb(V) removal was observed at acidic pH, i.e., 4 and 5, which may be attributable to the highly positive surface potential (around 40 mV) and reduced availability of FC precipitates. Interestingly, no Sb(V) removal was observed at alkaline pH (9 and 10) (Figure 1C), which might be related to the stabilization of FC precipitates due to high negative ζ-potential. Under these conditions, this is due to the increase in electrostatic repulsion between negatively charged Fe(OH)_4_^−^ and negatively charged Sb(OH)_6_^−^ species (Figure S1A,B). Our findings suggested that the redox Sb species may influence the ζ-potential, HDD and stability of FC precipitates, thereby affecting the overall Sb removal during the chemical coagulation process.

### 2.2. Dominant Mechanism Involved in Sb Removal

**Figure 2:**
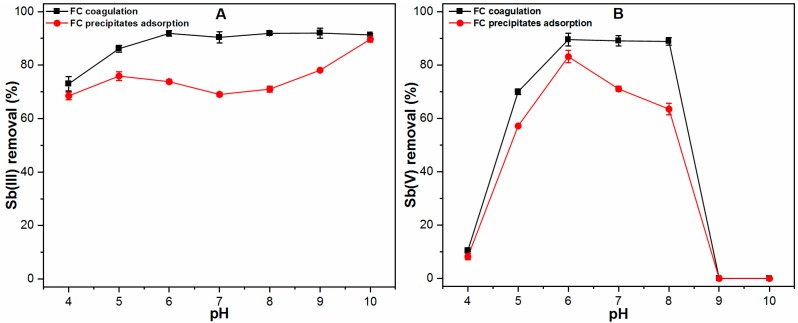
Comparison of (**A**) Sb(III) and; (**B**) Sb(V) removal via FC coagulation and adsorption onto preformed FC precipitates across a wide pH range (4–10).

The chemical coagulation process mainly involves several removal mechanisms including precipitation, co-precipitation and adsorption. As described previously, the possibility of precipitation can be ignored due to the fact that, if precipitation of Sb(III, V) species is occurring, the residual Sb concentration remains the same irrespective of the contaminant loading and other competing ions, in accordance with the principle of “constant solubility product” [10]. Therefore, it will be worth examining the relative percentage of co-precipitation and adsorption mechanisms involved during the chemical coagulation process. The Sb(III, V) removal as a function of pH was therefore investigated via Sb adsorption onto preformed FC precipitates and compared with Sb coagulation data in order to better elucidate the dominant removal mechanism (Figure 2).

The results indicated that the solution pH directly affects the relative importance of the removal mechanisms of redox Sb species. As shown in Figure 2A, the Sb(III) removal efficiencies via coagulation and adsorption onto preformed FC precipitates were similar at pH 4 and 10, thus indicating that adsorption was the dominant removal mechanism at these pH conditions. However, a noticeable difference via coagulation and adsorption was observed in Sb(III) removal efficiencies for the pH range of 5–9, indicating the contribution of co-precipitation mechanism under such a pH range. Co-precipitation is defined as the incorporation of soluble Sb(III, V) species into a growing hydroxide phase (Fe(OH)_3_). For instance, the substantial differences (co-precipitation) were observed in Sb(III) removal at pH 7 and 8 to be 21.37% and 20.89% respectively. The adsorptions of Sb(III) onto preformed FC precipitates were found to be 69.04% and 71.02%, still showing adsorption as a predominant coagulation mechanism (Figure 2A). Figure 2B presents the Sb(V) removal efficiencies via coagulation and adsorption across a wide pH range of 4–10. Similar to Sb(III), the significant differences in Sb(V) removal were observed at pH 7 and 8 to be 22.04% and 29.36% respectively, while the adsorption experiment indicated respective values of 67.03% and 59.48% at those pH conditions. Thus, the predominant mechanism involved in Sb(V) removal was still considered to be adsorption. These results suggested that the Sb(III, V) removal is mainly governed by the adsorption mechanism during the chemical coagulation process.

Previous studies [10,12,13,14,16,19] showed similarities with and differences from the proposed Sb(III, V) removal mechanism, as was shown in the current study. Guo et al. [10] compared the Sb(III, V) removal via FC coagulation with solid hydrous ferric oxide at pH 6. Their results suggested that adsorption is the main mechanism involved in Sb(III) removal; however, in the case of Sb(V) removal, other mechanisms also play a significant role in the FC coagulation process. Our previous studies [12,13] also described that, at pH 7, the main mechanism involved in Sb removal is the adsorption of contaminants onto FC precipitates. By contrast, some recent studies [14,19] suggested the involvement of a co-precipitation mechanism, namely Sb(III) anomalous incorporation, in facilitating Sb(III) removal by FC coagulation. A discrepancy remains in understanding the Sb(V) removal mechanism. Therefore, our current study indicates that both adsorption and co-precipitation are significant for Sb(III, V) removal at pH 7, while at acidic and alkaline pH, the adsorption of Sb species onto FC precipitate is the main removal mechanism of redox species. Our results are consistent with those of a previous study [17] which indicated the adsorption as a primary removal mechanism of As(III, V) species by the FC coagulation process.

### 2.3. Effect of Metal Cations on Physico-chemical Properties of Precipitates and Sb Removal

#### 2.3.1. Monovalent Cation

**Figure 3:**
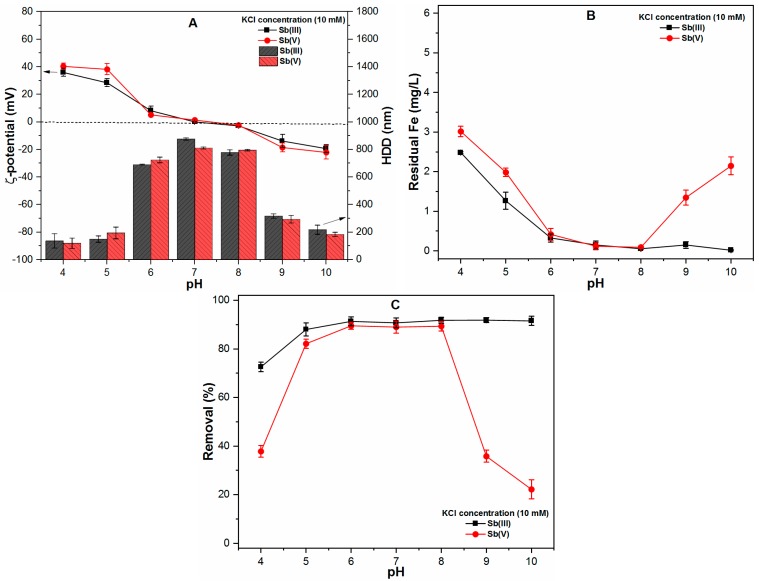
At various pH ranges (4–10), FC dose (0.1 mM), temperature (25 ± 1 °C), KCl concentration (10 mM) and Sb(III, V) concentration (1 mg/L) showing (**A**) ζ-potential (mV) and HDD of precipitates (nm); (**B**) Residual Fe (mg/L) and; (**C**) Removal (%) of both Sb species.

Figure 3 shows the effect of monovalent cation (KCl) on the properties of FC precipitates as well as their effects on Sb removal during the coagulation process. The effect of the low concentration of KCl (1 mM) was found to be insignificant on the overall performance (Supplementary: Figure S2), therefore, a high concentration of KCl (10 mM) was used in both the Sb(III) and Sb(V) systems. The results indicated that the HDD of precipitates increased throughout the studied pH range (4–10) (Figure 3A) when compared with control conditions (Figure 1A). The ζ-potential shifted towards more positive values at acidic pH (4 and 5) and more FC precipitation was observed at those pH values as well (Figure 3A,B). In the pH range of 6–8, a slight reduction was observed in the magnitude of ζ-potential, along with larger size precipitates (Figure 3A). Moreover, at pH 9 and 10, the ζ-potential approached pzc, so more FC precipitation was observed, particularly in the case of the Sb(V) system (Figure 3A,B). This might be related to the electrical double layer (EDL) compression, which increases the collision frequencies between the colloidal precipitates, thereby reducing the stability of the suspension. This led to the formation of larger size precipitates and a drastically decreased Fe solubility in solution [17]. In comparison, the insignificant difference was observed in Sb(III) removal under these conditions; however, Sb(V) removal was enhanced at acidic and alkaline conditions in the presence of 10 mM KCl (Figure 3C). For example, at pH 4 and 10, the removals of Sb(V) were observed to be 37.82% and 22.20%, respectively (Figure 3C), which is greater than the values of 10.43% and 0% observed in the absence of monovalent cation (Figure 1C). These results suggested that a high concentration of monovalent cation may enhance the HDD of precipitates and also increase the Sb(V) removal efficiencies at acidic and alkaline conditions during the chemical coagulation process.

#### 2.3.2. Divalent Cation

**Figure 4:**
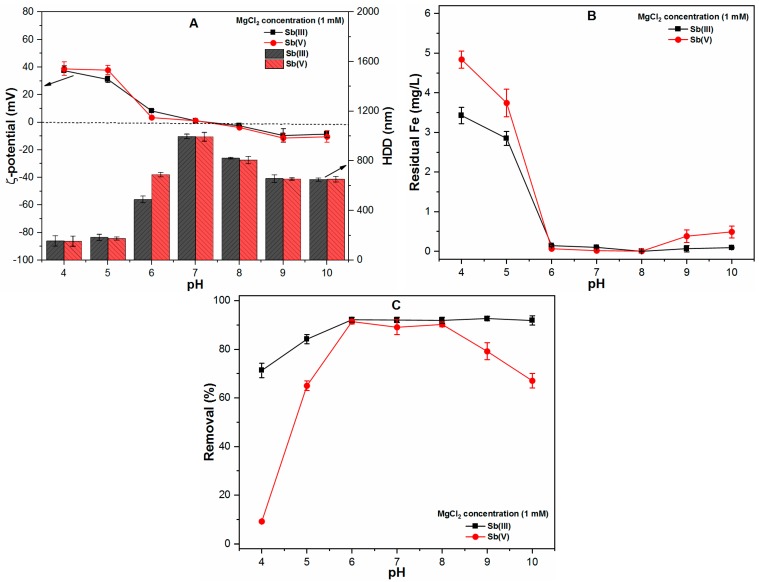
At various pH ranges (4–10), FC dose (0.1 mM), temperature (25 ± 1 °C), MgCl_2_ concentration (1 mM) and Sb(III, V) concentration (1 mg/L) showing (**A**) ζ-potential (mV) and HDD of precipitates (nm); (**B**) Residual Fe (mg/L) and; (**C**) Removal (%) of both Sb species.

The divalent cations are ubiquitous in natural water sources, particularly in groundwater. Therefore, the effect of MgCl_2_ (1 mM) on the properties of precipitates and Sb(III, V) removal was evaluated by FC coagulation (Figure 4). For the pH range of 4–6, marginal changes were observed in the ζ-potential; however, at alkaline pH (9–10), the ζ-potential values approached pzc and as a result, the HDD was significantly increased to around 650 nm in both the Sb(III) and Sb(V) systems (Figure 4A). It may also be noted that the residual Fe concentration was significantly decreased at alkaline pH conditions, particularly in the case of the Sb(V) system (Figure 4B). The divalent cations effectively compress the EDL of FC precipitates in the solution. Moreover, it is also involved in the co-precipitation process with ferric species, thereby increasing the HDD of precipitates and thus leading to decreased FC solubility under these conditions [20,21].

Figure 4C presents the removal efficiencies of Sb(III, V) species in the presence of a divalent cation. The results indicated that the presence of a low concentration of MgCl_2_ (1 mM) has an insignificant effect on Sb(III) removal. However, compared to monovalent cation, the divalent cation significantly improves the removal of Sb(V) at alkaline pH (9 and 10) to 79.16% and 67.06%, respectively (Figure 4C). Such removal behavior occurs parallel to the increased HDD of precipitates and enhanced FC precipitation in the Sb(V) system (Figure 4A,B). Similarly, previous studies [22,23] also reported the improvement in As(V) removal efficiency at alkaline pH under the influence of divalent cations.

### 2.4. Effect of Organic Matter on Physico-chemical Properties of Precipitates and Sb Removal

**Figure 5:**
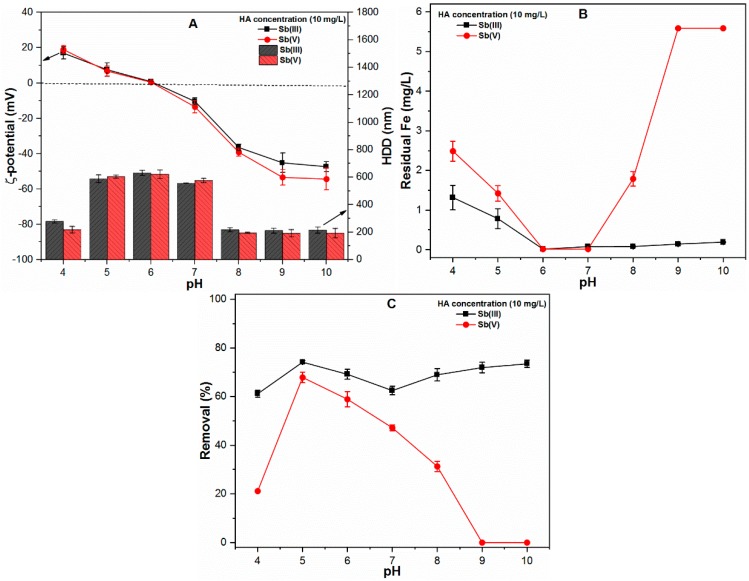
At various pH ranges (4–10), FC dose (0.1 mM), temperature (25 ± 1 °C), HA concentration (10 mg/L) and Sb(III, V) concentration (1 mg/L) showing (**A**) ζ-potential (mV) and HDD of precipitates (nm); (**B**) Residual Fe (mg/L) and; (**C**) Removal (%) of both Sb species.

The presence of organic matter such as HA in Sb-rich water may affect the FC coagulation performance. Therefore, the experiments were conducted to evaluate the properties and stability of precipitates as well as their impact on Sb removal under the influence of HA (10 mg/L) molecules (Figure 5). Moreover, the ζ-potential of HA (10 mg/L) as a function of pH had also been presented in Figure S3. The results indicated that the negatively charged HA molecules bring the ζ-potential close to pzc at acidic pH (4 and 5) and shifts the pzc to pH 6, thereby presenting the largest HDD of coagulated precipitates at pH 6 (Figure 5A). Therefore, the residual Fe concentration was reduced under acidic pH conditions (Figure 5B). Significant decreases in the HDD of precipitates were observed at pH 7 and 8, which may be attributed to the shift of ζ-potential values, specifically up to around −12 mV and −38 mV in both Sb(III) and Sb(V) systems (Figure 5A). Moreover, high negative ζ-potential values were observed in the Sb(V) system at pH 9 (−53.42 mV) and 10 (−54.37 mV) (Figure 5A). Aside from the negligible effect on Fe solubility in the Sb(III) system, the Sb(III) removal was remarkably reduced in the presence of HA over the entire pH range (Figure 5B, C). This may be attributed to the fact that HA molecules have a high binding affinity for FC precipitates, modify FC surface properties (ζ-potential values and HDD of precipitates) and effectively block adsorption sites for Sb species [11,24,25]. The presence of HA significantly affects the Sb(V) removal across a wide pH range. In contrast to Sb(III), the removal efficiency of Sb(V) remains unchanged at pH 5, while it decreases substantially when the pH is increased from 6–10. For instance, at pH 6, 7 and 8, the Sb(V) removal efficiencies decreased by 30.63%, 41.90% and 57.55% respectively. These significant reductions in Sb(V) removal may be attributed to the decrease of ζ-potential values and hindrance of FC precipitation process in solution (Figure 5A,B). Moreover, HA molecules also form complexes with metal oxide surfaces, alter their surface properties, thus the competitive inhibition effect of HA molecules increases the mobility of Sb in aqueous solution [26,27]. In general, the presence of organic matter influences the properties as well as solubility of FC species in water and affects the overall performance of the chemical coagulation process.

## 3. Materials and Methods

### 3.1. Chemicals and Solutions Preparation

The reagent grade chemicals, antimony (III) oxide (Sb_2_O_3_); potassium hexahydro-antimonate (KSb(OH)_6_) and technical grade chemical, humic acid (HA) were obtained from Sigma-Aldrich (St. Louis, MO, USA). The analytical grade chemicals, ferric chloride hexahydrate (FeCl_3_.6H_2_O), potassium chloride (KCl), magnesium chloride (MgCl_2_), hydrochloric acid (HCl) and sodium hydroxide (NaOH), were purchased from Samchun (Samchun pure Chemicals Co., Ltd., Pyeongteak-si, Korea). The 100 mg/L Sb(III) and Sb(V) stock solutions were prepared by dissolving Sb_2_O_3_ and KSb(OH)_6_ in 2M HCl and deionized (DI) water, respectively. The FeCl_3_·6H_2_O (FC), KCl and MgCl_2_ were added into DI water to prepare the 0.1 M (27,029 mg/L) FC, 1 M KCl and 1 M MgCl_2_ stock solutions, respectively. The stock solution of model organic matter was prepared by dissolving 100 mg HA powder in 0.1 L DI water. The pH of the HA stock solution was adjusted to 11 using NaOH, then stirred at 600 rpm for 24 h and filtered using a 0.45 µm glass fiber filter. The pH of the HA stock solution was set to 7.0 ± 0.1 before being stored in the dark at 4 °C [11]. The equivalent total organic carbon (TOC) concentration for HA with 10 mg/L was also measured using a TOC analyzer with an ASI-L liquid autosampler (TOC-5000A, Shimadzu Corp, Kyoto, Japan) and it was found to be 4.17 mg C/L.

### 3.2. Chemical Coagulation Experiments

#### 3.2.1. Experimental Procedures

The chemical coagulation experiments were conducted using jar tester equipment (Model: SJ-10, Young Hana Tech Co., Ltd., Gyeongsangbuk-Do, Korea) with six blades at 25 ± 1 °C. The pH was adjusted to predetermined levels for each experiment using either 0.1 M HCl or 0.1 M NaOH solutions. The coagulation experiments were conducted for each of the experimental conditions according to the following three sequential steps: coagulation via rapid mixing at 140 rpm for 3 min, flocculation via slow mixing at 40 rpm for 20 min and quiescent settling for 30 min [11,12,13,28]. The sample was then collected to measure the ζ-potential and HDD of early formed FC precipitates. Upon completion of the jar test procedure, the aliquot was filtered using a 0.22 µm PES membrane to evaluate the residual concentrations of Fe and Sb species in the supernatant. In the adsorption experiments, the FC precipitates were initially formed by coagulating 0.1 mM FC in solution, then Sb species were added, then slow stirring and quiescent settling proceeded. The residual Sb concentrations were then analyzed to determine the adsorption of Sb on preformed FC precipitates.

#### 3.2.2. Experimental Conditions

For all of the chemical coagulation experiments, the experimental conditions were as follows: 1 mg/L Sb(III, V), optimum FC dose (0.1 mM or 27.029 mg/L), temperature (25 ± 1 °C) and a pH range of 4–10 [12,13]. The experiments were then separately conducted in the absence and presence of KCl (1 and 10 mM), MgCl_2_ (1 mM) and HA (10 mg/L) in order to explore the ζ-potential and HDD of precipitates, as well as to examine the residual Fe concentration and Sb(III, V) removal. Furthermore, a separate set of adsorption experiments was performed using preformed FC precipitates in the pH range of 4–10 to explore the dominant mechanism involved in Sb(III, V) removal. All experiments were performed in triplicate and the relative standard deviations (RSD) were reported.

### 3.3. Analytical Methods

The residual concentrations of the Fe and Sb species were analyzed through Inductively Coupled Plasma Optical Emission Spectrometry (ICP-OES: Model Varian, Agilent technologies, Sana Clara, CA, USA). The solution pH was set using a pH meter (HACH: HQ40d Portable pH, Conductivity, oxidation-reduction potential (ORP) and an ion selective electrode (ISE: Multi-Parameter Meter, Hach Company, Loveland, CO, USA). The ζ-potential and HDD of the precipitates were measured using a Zetasizer (Zeta-sizer, NanoZS, Malvern, Worcestershire, UK). The chemical modeling software Visual MINTEQ 3.1 (KTH, Stockholm, Sweden) was used to obtain the speciation diagrams of Fe(III), Sb(III) and Sb(V) species. In addition, the graphical software OriginPro 9.5 (OriginLab, Massachusetts, MA, USA) was used to plot the experimental data.

## 4. Conclusions

In this study, we systematically examined the properties and solubility of FC precipitates and their respective Sb(III, V) removal under the influence of various solution chemistries. Our results showed that the properties of precipitates had an insignificant effect on Sb(III) removal under the studied pH range, while it significantly influences the Sb(V) removal efficiencies at alkaline pH conditions. The adsorption was identified as a dominant removal mechanism for both Sb(III) and Sb(V) species. The HDD of precipitates showed an insignificant effect on the coagulation performance in both the Sb(III) and Sb(V) systems. Moreover, the FC precipitation was remarkably influenced in the Sb(V) system, particularly at alkaline pH conditions. The addition of divalent cation (MgCl_2_) shifts the ζ-potential of precipitates at alkaline pH in the Sb(V) system, thereby decreasing the Fe solubility and enhancing the Sb(V) removal. In both systems, the presence of humic acid decreases the ζ-potential and HDD of coagulated precipitates, thus influencing the FC precipitation and Sb removal during the coagulation process. These results suggested that the water characteristics may influence the properties of precipitates as well as the removal performance of Sb species during the water treatment process.

## Supplementary Materials

Supplementary materials can be found at https://www.mdpi.com/1422-0067/20/12/2945/s1. Figure S1. Speciation diagrams of (A) Fe(III); (B) Sb(V) and; (C) Sb(III) at 25 ± 1 °C derived using Visual MINTEQ 3.1; Figure S2. At various pH ranges (4–10), FC dose (0.1 mM), temperature (25 ± 1 °C), KCl concentration (1 mM) and Sb(III, V) concentration (1 mg/L) showing (A) ζ-potential (mV) and HDD of precipitates (nm); (B) Residual Fe (mg/L) and; (C) Removal (%) of both Sb species. Figure S3. ζ-potential of humic acid (10 mg/L) under different pH (4-10).

## Abbreviations

HDD	Hydrodynamic diameter
HA	Humic acid
DI	Deionized
RSD	Relative standard deviation
TOC	Total organic carbon
USEPA	United States Environmental Protection Agency
EU	European Union
WHO	World Health Organization
pzc	Point of zero charge
EDL	Electrical double layer
