# Supplementary material for: Effect of Water Chemistry on Antimony Removal by Chemical Coagulation: Implications of ζ-Potential and Size of Precipitates
Source: Int J Mol Sci. 2019 Jun 17;20(12):2945. doi: 10.3390/ijms20122945 (PMC6627716; doi:10.3390/ijms20122945)
Supplement: Supplementary file 1 [file ijms-20-02945-s001.pdf]

International Journal of
Molecular Sciences

MDPI

Supplementary Information

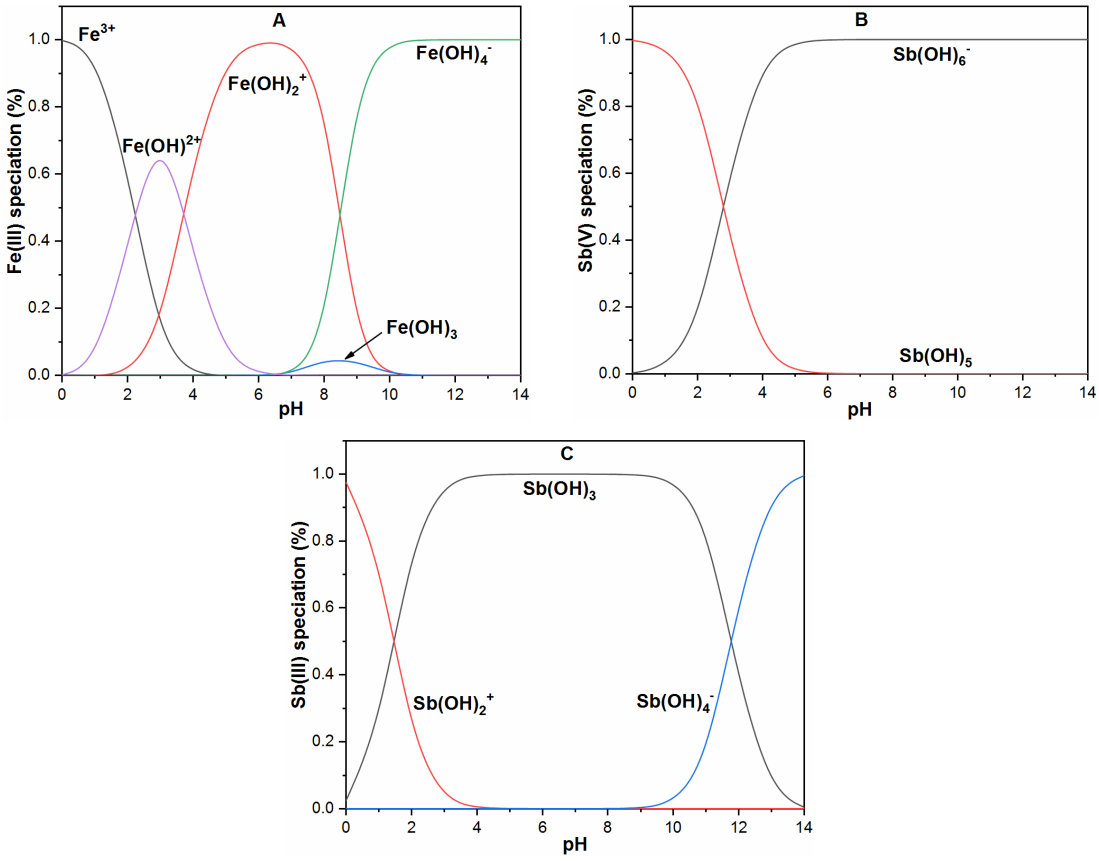

Figure S1. Speciation diagrams of (**A**) Fe(III); **(B)** Sb(V) and; **(C)** Sb(III) at 25 ± 1ºC derived using Visual MINTEQ 3.1.

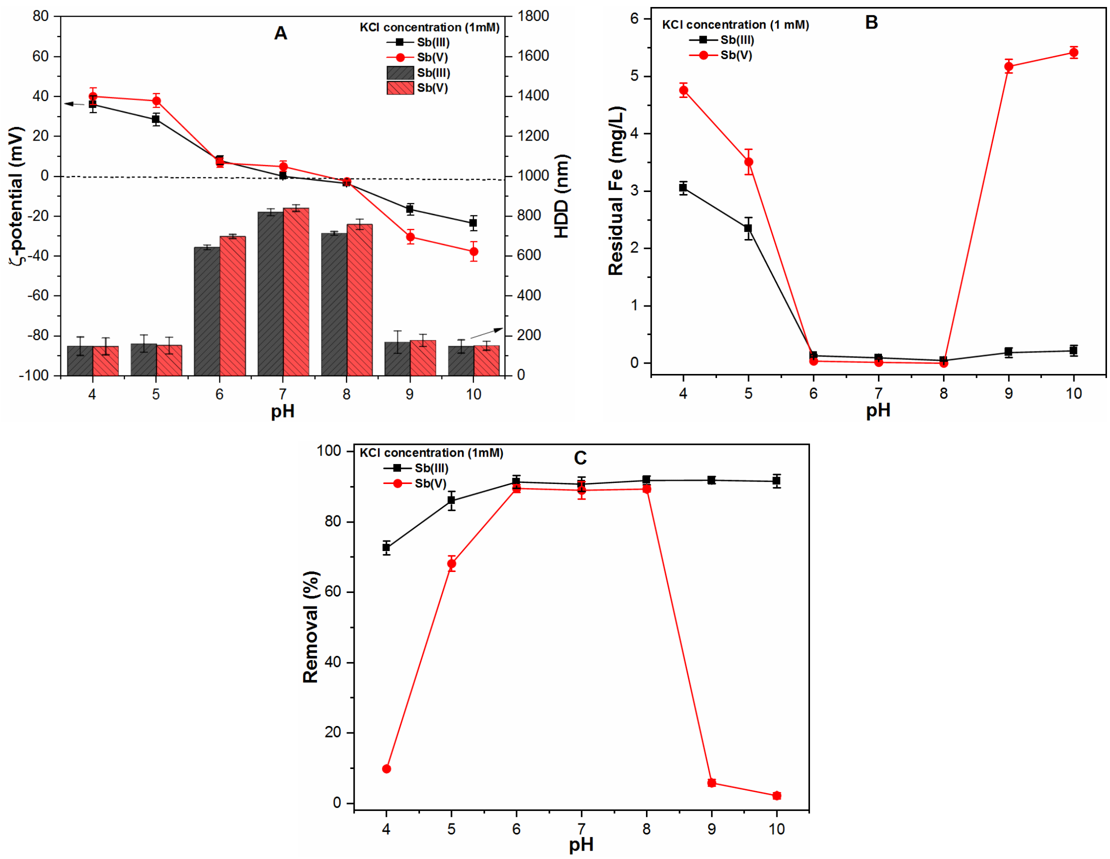

Figure S2. At various pH range (4-10), FC dose (0.1 mM), temperature (25 ± 1°C), KCl concentration (1 mM) and Sb(III, V) concentration (1 mg/L) showing **(A)** ζ-potential (mV) and HDD (nm) of precipitates; **(B)** Residual Fe (mg/L) and; **(C)** Removal (%) of both Sb species.

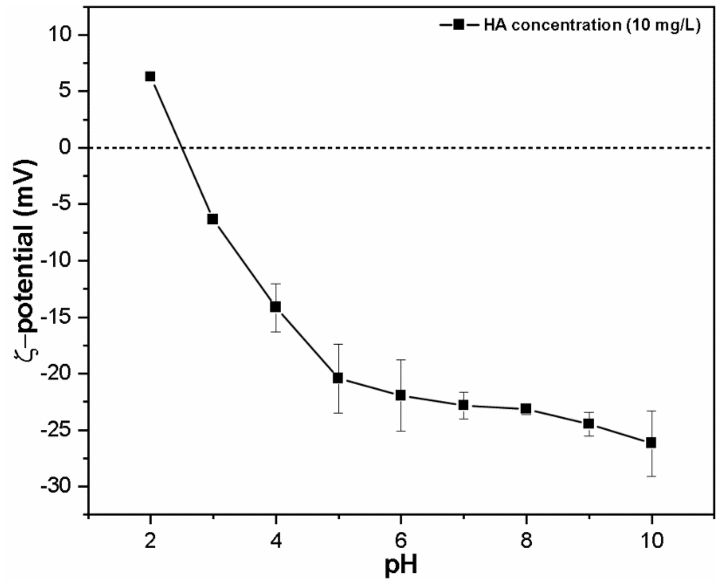

Figure S1. ζ-potential of humic acid (10 mg/L) under different pH (4-10).

© 2019 by the authors. Submitted for possible open access publication under the terms and conditions of the Creative Commons Attribution (CC BY) license (http://creativecommons.org/licenses/by/4.0/).
